# Antimicrobial stewardship programs in solid‐organ transplant recipients in Switzerland

**DOI:** 10.1111/tid.13902

**Published:** 2022-10-18

**Authors:** Julia A. Bielicki, Oriol Manuel, the Swiss Transplant Cohort Study (STCS)

**Affiliations:** ^1^ Department of Paediatric Pharmacology University of Basel Children's Hospital (UKBB) Basel Switzerland; ^2^ Department of Infectious Diseases and Vaccinology University of Basel Children's Hospital (UKBB) Basel Switzerland; ^3^ Infectious Diseases Service and Transplantation Center Lausanne University Hospital (CHUV) and University of Lausanne Lausanne Switzerland

**Keywords:** antibiotic use, antimicrobial stewardship, transplant infections

## Abstract

**Introduction:**

Antimicrobial stewardship programs (ASPs) are essential for minimizing the emergence of antimicrobial resistance, while improving patient outcomes. The current status of ASP in the field of organ transplantation in Switzerland has not been well characterized.

**Methods:**

We describe in this article the current status of ASP and discuss challenges and opportunities of implementing ASP dedicated to solid‐organ transplant (SOT) recipients in Switzerland.

**Results:**

ASP have been implemented in the Swiss healthcare system over the last years, although specific strategies for SOT recipients are mostly based on transplant infectious diseases (TID) consultations rather than structured institutional interventions. Even so, there is a unique opportunity for developing a successful ASP in Switzerland that also specifically addresses areas of practice relevant to SOT recipients. This is due to the existent network of TID specialists in close collaboration with transplant physicians, the small number of centers involved in the care of transplant recipients, and the development of the Swiss Transplant Cohort Study (STCS), a prospective nationwide cohort of SOT recipients in Switzerland. The STCS can identify actual challenges through the updated reports on the epidemiology on transplant infections, accurately monitor the impact of potential antimicrobial stewardship interventions, and represent an opportunity for nesting of pragmatic randomized controlled trials to address key questions about optimized antibiotic use for SOT recipients.

**Conclusions:**

Although ASP in SOT recipients rely more on specific TID consultations than in general antimicrobial stewardship teams, we identified several opportunities for the implementation of a successful ASP in Switzerland.

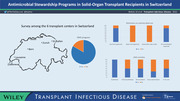

## 1 ORGAN TRANSPLANTATION IN SWITZERLAND

In Switzerland (population of 8.6 million inhabitants), organ transplantation is performed in five University Hospitals (Basel, Bern, Geneva, Lausanne, and Zurich) and one Cantonal Hospital (St. Gallen), comprising six kidney, three liver, three heart, two lung, and two pancreas transplant programs.^1^ Some of these programs are shared between University Hospitals. Transplant activity in Switzerland is stable, with a median of 580 transplants per year performed over the last 5 years. This includes a median of 345 kidney, 145 liver, 35 heart, 35 lung, and 20 pancreas transplantations per year. Although the number of transplant was dramatically reduced during the first wave of Covid‐19 (in particular for living donation), numbers reached to normal at the end of the year 2020 and are stable since.^2^ Swisstransplant, the national Swiss Organ Procurement Organization (OPO), maintains the organ‐recipient waiting list and allocates organs in accordance with Swiss legislation by coordinating a network of five autonomous regional OPOs.

Since 2008, data on all solid‐organ transplantations performed in Switzerland have been collected in a nationwide database named Swiss Transplant Cohort Study (STCS).^3^ This database has a twofold objective: on the one side to serve as a register of transplant activities and to summarize the main outcomes related with transplantation (minimal dataset). This registry is in accordance with legal regulations and requirements of the Federal Office of Public Health. On the other side, in patients consenting to participate in the STCS, a more detailed dataset (including infectious complications) and a biobank are collected. This extended dataset is used mainly for scientific purposes, and it is funded by the Swiss National Science Foundation.

Each transplant center has local referent persons in transplant infectious diseases (TID), organized through the Infectious Diseases Study Group of the STCS (for scientific questions), and through the Infectious Diseases Working Group (STAI) of Swisstransplant (for infectious diseases matters related with organ donation). This active network of TID specialist implements and coordinates research projects within the STCS, participates in the writing of national guidelines on the screening of infectious diseases in potential donors and recipients, guides transplant clinicians for the acceptance of infected organ donors, and discusses specific cases of difficult‐to‐treat infections in solid‐organ transplant (SOT) recipients.

## 2 EPIDEMIOLOGY OF TRANSPLANT‐INFECTIOUS DISEASES IN SWITZERLAND

The epidemiology and outcomes of transplant infections have been characterized in detail in several publications of the STCS. Some studies have described the general epidemiology of infection in all patients included in the cohort,^4^ whereas others have focused on a specific type of pathogen (e.g., cytomegalovirus and other herpesviruses,^5^, ^6^ *Aspergillus*,^7^ *Clostridioides difficile* ^8^) or on a specific syndrome (e.g., food‐borne infection,^9^ urinary tract infection^10^). In addition to the scientific value, these publications have a pivotal role in quantifying the burden of disease associated with infection, in describing local trends on the epidemiology of specific infections, and in identifying potential unexpected infectious diseases concerns in a particular center.^11^ Given the updated annual reports generated by the STCS, these potential concerns can be identified and addressed in a real‐time matter.

The most comprehensive data on the epidemiology of transplant infection in Switzerland has been summarized by van Delden et al.^4^ This study collected more than 3500 clinically significant infections occurring in more than 2700 SOT recipients during the first year posttransplant. Although opportunistic infections were rarely observed in the era of effective prophylaxis, bacterial infection represented about two thirds of all infections, being Enterobacterales and *Enterococcus* the most prevalent pathogens. Viruses and fungal pathogens caused 30% and 8% of infections, respectively. Up to 23% of *Pseudomonas aeruginosa* isolates were multidrug resistant, and 15% of *Klebsiella* spp. and *Escherichia coli* produced extended‐spectrum β‐lactamase (ESBL), but no infection due to carbapenemase‐producing bacteria was identified. A subsequent publication detailed the epidemiology and risk factors of ESBL‐producing Enterobacterales.^12^ Out of 1212 Enterobacterales infections, 138 (11%) were ESBL‐producing. The main risk factor for acquisition of ESBL‐producing Enterobacterales infection was a previous antibiotic exposure (aOR = 2.6, 95%‐CI: 1.0–6.8). Inappropriate antibiotic therapy has been associated with unfavorable outcomes, highlighting the need for specific antimicrobial stewardship programs (ASPs) in this population. Regarding enterococci infections, prevalence of vancomycin resistance was inexistent in an early study,^13^ although some cases have been observed since then in the context of hospital outbreaks. The burden of disease of vancomycin‐resistant enterococci and methicillin‐resistant *Staphylococcus aureus* in SOT recipients in Switzerland is currently being investigated. Other important published data that might help in delineating ASPs in SOT recipients is regarding *C. difficile*. Incidence of *C. difficile* infection from 2008 and 2013 was 4%, with a higher incidence in lung transplant as compared to kidney transplant recipients.^8^ Use of antibiotics during the 3 months before infection was the most robust risk factor for *C. difficile* infection (OR 4.51, 95% CI 2.03–10.00), in particular the use of carbapenems and quinolones. Importantly, *C. difficile* infection was associated with an increased risk for graft loss.

## 3 ANTIMICROBIAL USE IN SOT RECIPIENTS

The preventive and therapeutic strategies against infectious diseases in Switzerland vary according to organ and transplant center. Pre‐transplant antibacterial prophylaxis is frequently tailored to colonizing bacteria and not extended beyond 72‐h post‐surgery. Use of trimethoprim–sulfamethoxazole (TMP–SMX) prophylaxis is almost universal in lung transplant recipients, whereas it is administered in about two thirds of liver transplant recipients depending on a case‐specific risk assessment.^4^ Duration of TMP–SMX prophylaxis also varies among types of organs, with more than 80% of lung transplantation receiving TMP–SMX for more than 12 months, as compared to less than 20% in kidney transplant recipients. Other types of antibacterial prophylaxis are almost exclusively seen in lung transplant recipients. Antiviral prophylaxis is used in ∼60% of SOT recipients for 3–6 months, except again for lung transplant recipients where it is used in all patients for at least 6–12 months. Antifungal prophylaxis is used mostly in lung transplant recipients.^4^ Specific use of wide spectrum antimicrobials in SOT recipients is not systematically collected in the STCS.

## 4 CONTEXT OF ANTIMICROBIAL STEWARDSHIP IN SWITZERLAND

Antimicrobial stewardship is defined as a coherent set of activities adapted to a specific context that encourage the judicious use of antimicrobials.^14^ Although the minimization of the emergence of antimicrobial resistance is a key goal of antimicrobial stewardship, it is also considered essential to improve patient outcomes.^15^ Structured and consistent approaches to improving the use of antimicrobials are especially important for patients at high risk of invasive and difficult‐to‐treat infections, and more targeted utilization of antimicrobials has not been found to be associated with adverse outcomes in hemato‐oncological patients.^16^, ^17^ ASP for SOT recipients are challenging due to the added diagnostic and therapeutic uncertainties in this group compared to other patients receiving antimicrobial treatments.^18^

Switzerland, like many other countries, has several interrelated national strategies in place that address antimicrobial stewardship. The Strategy on Antibiotic Resistance (StAR) represents the Swiss National Action Plan on antimicrobial resistance.^19^ It takes a One Health approach, with all aspects of antimicrobial stewardship, such as availability of guidelines and strengthened surveillance, at the core of the response to antimicrobial resistance in the human sector. A relevant related national strategy is the NOSO strategy, which addresses the prevention and management of healthcare‐associated infections in acute care facilities in Switzerland.^20^ The cross section of a focus on healthcare‐associated infections and optimized antimicrobial use in acute care facilities is an obvious area of synergy between these two strategies.

The Swiss healthcare system is highly devolved, with much of the strategic and operational decision‐making about healthcare taking place at the cantonal (regional) level.^21^ A direct uniform implementation of antimicrobial stewardship policies across all parts of healthcare is therefore challenging. To facilitate coordinated implementation and joint monitoring, and to identify opportunities for knowledge transfer and shared learning, the National Centre for Infection Prevention (Swissnoso) is facilitating the development of a national Swiss Antimicrobial Stewardship Platform (SwissASP).^22^ SwissASP activities are coordinated by Swissnoso during the buildup phase with essential contributions from key national professional organizations and societies, including the Swiss Society for Infectious Diseases, the Swiss Society for Microbiology, and the Swiss Association of Public Health Administration and Hospital Pharmacists, as well as from the Swiss Center for Antibiotic Resistance (ANRESIS).

The overarching goal of SwissASP is to enable national action through engagement of experts, key stakeholders at a national level and representatives from acute care facilities who are involved in designing and delivering antimicrobial stewardship at their site. Ultimately, SwissASP will host a network of antimicrobial stewardship experts, coordinate national initiatives in this area of healthcare practice, and inform national implementation of antimicrobial stewardship policies as outlined in the StAR and NOSO strategies.

## 5 CURRENT STATE OF ANTIMICROBIAL STEWARDSHIP IN SWITZERLAND

To provide a common framework for delivering antimicrobial stewardship in Swiss acute care facilities, including for at‐risk groups such as SOT patients, SwissASP has published guidance on areas of action to be considered by healthcare facilities while reviewing the structural and implementation maturity of antimicrobial stewardship activities at their site. In a subsequent phase, an integrated approach toward monitoring of antimicrobial stewardship in acute care facilities, consisting, for example, of antimicrobial use surveillance, assessment of antimicrobial stewardship maturity, and tracking of nationally agreed quality indicators, will be developed. All monitoring approaches will include the possibility of monitoring site performance over time and benchmarking.

In a survey conducted at the initiation of the SwissASP activities, only a minority of responding hospitals indicated that a written strategy document or policy was in place for antimicrobial stewardship (21/63, 33% and 14/63, 22%, respectively).^23^ Instead, healthcare facilities were relying on antimicrobial treatment recommendations focusing on specific indications, surgical antibiotic prophylaxis, guidance on the avoidance of unnecessary broader‐spectrum antimicrobials, and duration of treatment.^23^ Approaches to the review of antimicrobial therapy based on clinical and diagnostic information, therapeutic drug monitoring, antifungal and antiviral treatment, and appropriate documentation around antimicrobial therapy were addressed only infrequently by hospital guidelines.^23^ Many of these aspects would be particularly relevant in a vulnerable patient group, such as SOT recipients.

## 6 ANTIMICROBIAL STEWARDSHIP FOR SOT RECIPIENTS

As mentioned, only six centers in Switzerland are considered primary SOT facilities. The leading infectious diseases physicians at those centers were approached to gather information on the current state of implementation of antimicrobial stewardship for SOT recipients (Table 1). Although restrictions for any antimicrobials, including requirements for up‐front approval by an antimicrobial stewardship team or infectious diseases expert, were being used in only 29% of responding hospitals in the 2016 national survey, all centers managing SOT recipients used some form of restriction.^23^ Similar to stewardship measures among general acute care facilities, there is a strong reliance on hospital guidelines to inform the management of infections in this patient group. Other activities are variably used; in particular antimicrobial cycling, antimicrobial order forms, and automatic alerts for unnecessarily duplicative therapy are not reported as being in use in any of the centers. Guideline coverage varies, with all centers having formal guidance on surgical perioperative antimicrobial prophylaxis in place, but only HAP/VAP (*n* = 3), bloodstream infection (*n* = 3), and *C. difficile* (*n* = 2) treatment guidelines being available in more than one center. Formal involvement of antimicrobial stewardship teams in the management of SOT patients is generally absent, probably related to the fact that strong expertise is directly provided by TID specialists in addressing these issues. In‐line with the general 2016 survey, there is limited or no auditing of specific antimicrobial management issues to identify adherence to guidelines and areas for quality improvement.^23^

**TABLE 1:** Survey on antimicrobial stewardship activities in transplant centers in Switzerland

Center	1	2	3	4	5	6
Number of SOT/year	50–150	50–150	<50	>150	50–150	50–150
Transplant ID consult service	24/7	24/7	24/7	24/7	24/7	24/7
Formal hospital AS program	No	Yes	Yes	Yes	Yes	Yes
Annual hospital cumulative antibiogram	Yes	Yes	No	Yes	Yes	Yes
Restrictions on antimicrobial use for SOT recipients						
Up‐front approval	Yes	Yes	Yes	Yes	Yes	No
Back‐end approval	No	No	No	No	No	No
Automatic stop order	No	No	No	No	No	No
ID consult required	Yes	No	Yes	Yes	Yes	Yes
Antimicrobial stewardship activities used, including for management of SOT recipients^a^						
Guidelines	Yes	Yes	Yes	Yes	Yes	Yes
Clinical pathways	No	No	Yes	Yes	Yes	No
Streamlining/de‐escalation	Yes	No	No	No	Yes	Yes
Dose optimization/adjustment	No	No	No	Yes	No	Yes
IV to oral conversion	Yes	No	No	Yes	No	Yes

Abbreviations: AS, antimicrobial stewardship; ID, infectious diseases; IV, intravenous; SOT, solid‐organ transplant.

^a^Note that the following activities were not reported as in use for SOT recipients in any of the responding centers: antimicrobial cycling, antimicrobial order forms, automatic alerts for unnecessarily duplicative therapy, and closed formulary.

## 7 OPPORTUNITIES AND CHALLENGES FOR ANTIMICROBIAL STEWARDSHIP FOR SOT RECIPIENTS

The reported activities in the Swiss transplant centers, including restrictions on antimicrobials and reliance on guidelines, and a focus on recommendations in the area of overlap between infectious diseases consults and antimicrobial stewardship (such as dose optimization, de‐escalation and intravenous to oral conversions), align with those known to be predominantly applied as part of antimicrobial stewardship for SOT recipients in other countries.^24^ The challenges of antimicrobial stewardship relate to the lack of robust evidence for key aspects of infection management in immunosuppressed patients, including SOT recipients.^24^ As an example, the duration of treatment for certain infections is often less well defined than in immunocompetent patients, making it difficult to use time‐sensitive stop orders.^25^ Thus, improvement of antimicrobial prescriptions is mostly based on specific transplant‐infectious diseases consultations rather than on institutional general rules for antimicrobial stewardship. This close involvement of infectious diseases specialist in the care of SOT recipients in Switzerland represents an opportunity for the development of ASPs specifically for this population. Engagement of SOT centers in an early stage of the development of SwissASP could mean that needs of SOT (and also stem‐cell transplant) recipients can be specifically addressed, for example, through a small working group within the platform that can be consulted on aspects relevant to these patient groups. Additionally, the small number of centers involved could work toward nationally aligned guidance on antimicrobial stewardship for SOT patients, including on use of diagnostics and prophylaxis. Specific interventions where more robust evidence is available for SOT recipients could be implemented initially, based also on local epidemiology and in collaboration with transplant specialists. Examples of targeted interventions can be a comprehensive program for reducing antibiotic use in kidney transplant recipients with asymptomatic bacteriuria,^26^ shortening the duration of antibiotic therapy for urinary tract infection,^27^ or apply standardized preventive strategies against invasive fungal infections in lung transplant recipients. In parallel, some challenges exist on the development of a successful stewardship program specifically for SOT recipients in Switzerland, mainly due to the highly devolved nature of the Swiss healthcare system, which means that any resource implications must be negotiated at a cantonal level.

## 8 CONCLUSIONS

In Switzerland, there is a unique opportunity for developing a successful ASP that also specifically addresses areas of practice relevant to SOT recipients. First, the implementation of such ASPs is a priority for the Swiss healthcare system, for which the structure and expertise in a national level is already in place, as previously mentioned. Second, the existent network of TID specialists, in close collaboration with transplant physicians, can facilitate the introduction of a targeted stewardship program. This task is easier to perform given the small number of Swiss transplant centers, and it can be based on the actual challenges identified through the updated reports on the epidemiology on transplant infections provided by the STCS. The STCS additionally allows one to accurately monitor the adherence to and impact of potential antimicrobial stewardship interventions. Lastly, the cohort could represent an opportunity for nesting of pragmatic randomized controlled trials to address key questions about optimized antibiotic use for SOT recipients, harnessing the close integration of infectious diseases and transplant services to generate a learning healthcare system.

## CONFLICT OF INTEREST

The authors have no conflicts of interest to declare. Please refer to the accompanying ICMJE disclosure forms for further details.

## FUNDING INFORMATION

The authors received no financial support to produce this manuscript.

## AUTHOR CONTRIBUTIONS

JAB and OM equally contributed to the implementation of the survey, revision of the literature, and writing of the manuscript.

## Supporting information

Graphical Abstract
