# Supplementary figures and images for: Antimicrobial stewardship programs in solid‐organ transplant recipients in Switzerland
Source: Transpl Infect Dis. 2022 Oct 18;24(5):e13902. doi: 10.1111/tid.13902 (PMC9788035; doi:10.1111/tid.13902)

**Visual abstract**


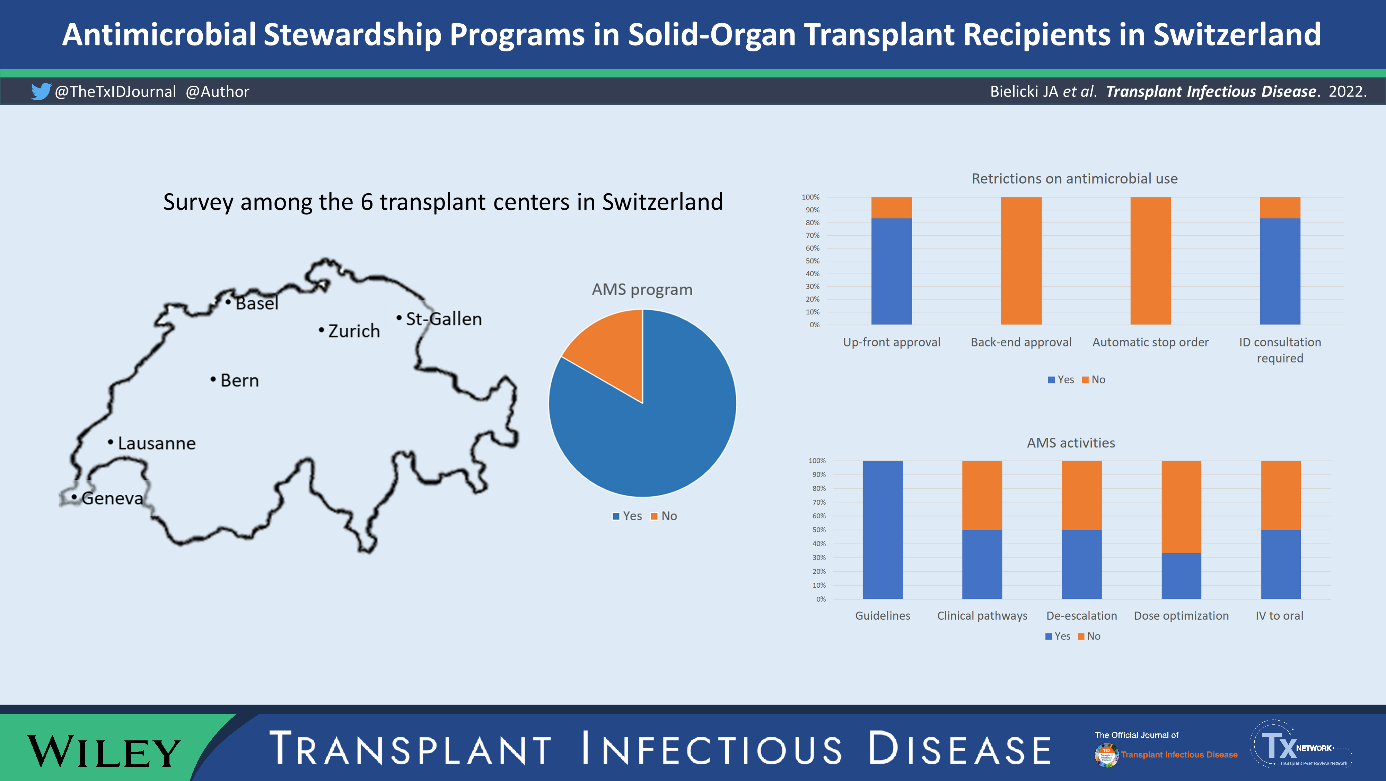

Supplement: Supplementary file 1 — Graphical Abstract [file TID-24-e13902-s001.docx]
